# Testing Stem Cell Therapy in a Rat Model of Inflammatory Bowel Disease: Role of Bone Marrow Stem Cells and Stem Cell Factor in Mucosal Regeneration

**DOI:** 10.1371/journal.pone.0107891

**Published:** 2014-10-13

**Authors:** Bo Qu, Guo-Rong Xin, Li-Xia Zhao, Hui Xing, Li-Ying Lian, Hai-Yan Jiang, Jia-Zhao Tong, Bei-Bei Wang, Shi-Zhu Jin

**Affiliations:** 1 Department of Gastroenterology and Hepatology, The Second Affiliated Hospital, Harbin Medical University, Harbin, China; 2 Anorectal Department, The First Affiliated Hospital, JiaMuSi Medical University, JiaMuSi, China; 3 Infections Department, JiaMuSi Central Hospital, JiaMuSi, China; University Claude Bernard Lyon 1, France

## Abstract

**Background:**

The gastrointestinal (GI) mucosal cells turnover regularly under physiological conditions, which may be stimulated in various pathological situations including inflammation. Local epithelial stem cells appear to play a major role in such mucosal renewal or pathological regeneration. Less is clear about the involvement of multipotent stem cells from blood in GI repair. We attempted to explore a role of bone marrow mesenchymal stromal cells (BMMSCs) and soluble stem cell factor (SCF) in GI mucosa regeneration in a rat model of inflammatory bowel diseases (IBD).

**Methods:**

BMMSCs labelled with the fluorescent dye PKH26 from donor rats were transfused into rats suffering indomethacin-induced GI injury. Experimental effects by BMMSCs transplant and SCF were determined by morphometry of intestinal mucosa, double labeling of PKH26 positive BMMSCs with endogenous proliferative and intestinal cell markers, and western blot and PCR analyses of the above molecular markers in the recipient rats relative to controls.

**Results:**

PKH26 positive BMMSCs were found in the recipient mucosa, partially colocalizing with the proliferating cell nuclear antigen (PCNA), Lgr5, Musashi-1 and ephrin-B3. mRNA and protein levels of PCNA, Lgr5, Musashi-1 and ephrin-B3 were elevated in the intestine in BMMSCs-treated rats, most prominent in the BMMSCs-SCF co-treatment group. The mucosal layer and the crypt layer of the small intestine were thicker in BMMSCs-treated rats, more evident in the BMMSCs-SCF co-treatment group.

**Conclusion:**

BMMSCs and SCF participate in but may play a synergistic role in mucosal cell regeneration following experimentally induced intestinal injury. Bone marrow stem cell therapy and SCF administration may be of therapeutic value in IBD.

## Introduction

Inflammatory bowel diseases (IBD) are common gastroenterological (GI) disorders among both the paediatric and adult populations [1]. This group of diseases is featured by a chronic, relapsing or remitting course of GI complaints such as diarrhea, rectal bleeding and abdominal cramping, which can result in malnutrition, weakness, electrolyte imbalances, and delayed growth, especially in children [2]. Histopathlogically, inflammation, cell degeneration, ulceration and fistulation may be found in the small and large intestines. Notably, excessive cellular regeneration appears also a part of the chronic pathogenic process, manifested as activation/proliferation of crypt cells and angiogenesis [3]. The etiology and pathogenic mechanism underlying IBD remain largely elusive. Autoimmunity may play a central role in the pathogenic interplay between genetic predisposition, microbial infection and environmental insults [4], [5]. Serological and other non-gastrointestinal signatures characteristic of autoimmune attack are commonly seen in IBD, especially evident in ulcerative colitis and Crohn's disease [6].

The mucosa of the GI tract undergoes physiological turnover occurring in every 2–7 days, which may be enhanced under pathological conditions [7]. Polipotent precursors located at the crypts are considered the major reservoir for the normal cellular renewal or pathological regeneration. These local stem cells may proliferate and differentiate into epithelium and glandulous cells in the mucosa [8]. Notably, recent studies suggest that bone marrow mesenchymal stromal cells (BMMSCs) may participate in GI regeneration especially under pathological conditions, including in IBD [9]. However, less is clear about the differentiation and early integration of BMMSCs in injured intestine. A better understanding of the role of blood-derived stem cells and soluble factors in GI histological and functional repair may shed new light on clinical management of IBD. In the present study we isolated BMMSCs from adult rats, pre-labelled them with a lipophilic red fluorescence dye PKH26. These labelled BMMSCs were then transplanted into recipient rats that suffered GI injury induced by indomethacin, an experimental model of IBD. The involvement of transplanted BMMSCs in cellular repair of the injured GI tract was studied by morphological and biochemical approaches. A potential synergetic therapeutic benefit by BMMSCs transplant and stem cell factor (SCF) administration was explored using our model system.

## Materials and Methods

### Animals and indomethacin treatment

Male Sprague-Dawley rats aged at 2 months were purchased from the Animal facility of Second Affiliated Hospital of Harbin Medical University. Animals were housed at constant temperature and humidity, with a 12/12 hr light/dark illumination cycle and free access to food chow and water. All experimental procedures were approved by the Harbin Medical University Administrative Panel on Laboratory Animal Care.

The experimental model of IBD was established by indomethacin administration in adult rats according to previous reports [10]. The animals were treated by subcutaneous injections of indomethacin (7.5 mg/kg in 5% sodium bicarbonate) 24 hours apart for 3 consecutive days. Four indomethacin-treated rats were killed under anesthetized condition with pentobarbital (50 mg/kg, i.p.) to evaluate the establishment of IBD model with histology. Additional animals (n = 18) were divided into 4 groups: (1) Receiving BMMSCs transplant and SCF treatment (BMMSCs-SCF group, n = 16); (2) receiving BMMSCs transplant (BMMSCs group, n = 16); (3) receiving SCF treatment (SCF group, n = 16); and (4) vehicle control with saline infusion (saline group, n = 16). The experimental effects were examined at day 7, 14, 21 and 28 days after BMMSCs, SCF or saline treatment.

### BMMSCs isolation, cultivation and fluorescent pre-labeling

BMMSCs isolation, cultivation and fluorescent pre-labeling were carried out according to previously described methods [11]. Briefly, 2 month-old adult rats (n = 4, also served as naive controls) were anesthetized by sodium pentobarbital (50 mg/kg, i.p.), and the femoral bones were removed. The medullar cavity was bathed by heparin (50 U/mL) in normal saline, and the marrow was aspirated and suspended in a lymphocyte isolation medium, followed by centrifuge at 2000 rpm for 20 minutes. The cell pellets were subsequently diluted with DMEM/F12 medium (DMEM/F12, 15% FBS, 100,000 U/L penicillin, pH = 7.4) to yield a density of more than 5×10^7^ cells/mL. Cells were collected after centrifuging at 1000 rpm for 10 minutes. BMMSCs were expanded *in vitro* after 10–15 days of culture. The cultured cells were then pre-labelled by the red-fluorescent lipophilic dye PKH26 according to the manufacturer's instruction [12]. The density of PKH26-labeled cell suspension was adjusted to approximately 3×10^7^ cells/mL. Subsequently, the harvested cells with viability greater than 95% measured by trypan blue exclusion were used for cell transplant studies.

### Fluorescence-activated cell sorting analysis

Samples of cultured cells were used for fluorescence-activated cell sorting (FACS) analysis in order to determine the expression of various signature antigen markers of mesenchymal stem cells. Approximately 1×10^6^ cells were incubated in 2% fetal bovine serum in 0.01 M phosphate-buffered saline (PBS, pH = 7.2) at 4°C for 30 minutes with 1 µl of monoclonal antibody specific for CD29 (BioLegend, 555005), CD34 (Santa Cruz, sc-7324), CD44 (BioLegend, 550947), CD45 (BioLegend, 554878), CD90 (BioLegend, 561409). Negative control was processed by incubating the cells in buffer without primary antibodies. The immunofluorescent signal was analyzed using the FACS Calibur with CellQuest software (Becton Dickinson, USA).

### Tissue preparation

Animals were perfused via the ascending aorta with PBS under overdose aesthesia (sodium pentobarbital 100 mg/kg, i.p.). The entire small intestine, appendix and colon were removed from the abdominal cavity for visual inspection and tissue sampling. Four small intestine segments, each approximately 2 mm in length, were blocked immediately proximal to the appendix, and further proximally to it, at approximately every 20 cm intervals. These intestine segments were rinsed in cold PBS for several times to remove contents, and then immersed in 4% paraformaldehyde for 24 hours. The former intestinal samples were embedded in paraffin, sectioned at 6 µm thickness, and used for histology and immunohistochemistry. For each animal case, at least one set of equally-spaced (approximately 60 µm in part) paraffin sections (10 sections mounted on one microslide) was used for a given histological or immunohistochemical stain. The segment left between the first and second sampled parts of the above was snap-frozen in liquid nitrogen for western blot and polymerase chain reaction (PCR) assays.

### Histology and Immunohistochemistry

Consecutive intestine sections were stained with H&E and immunoflourescence. Sections were de-waxed, and rehydrated after descending ethanol incubations prior to staining. PKH26-labeled cells in the intestine were examined with and without immunofluorescent colabeling. For double fluorescent labeling, dewaxed sections were preincubated in PBS containing 5% normal donkey serum for 30 minutes. followed by incubation with mouse anti-proliferating cell nuclear antigen (PCNA) (Chemicon, MAB424R, 1∶1000), rabbit anti-Lgr5 (Sigma-Aldrich-China, HPA012530, 1∶400), rabbit anti-Msi-1 (Sigma-Aldrich-China, SAB4200581, 1∶400) and eprhin-B3 (Abcam, ab101699, 1∶200) antibodies, respectively, at 4°C overnight. The specific immunoreactive signal was visualized by further incubation with DyLight 488 goat anti-mouse or rabbit IgG (H+L) (1∶200, Earthox, E032220) for 1 hr at room temperature. Sections were then counterstained with DAPI, washed and mounted with anti-fading medium before microscopic examination using a laser confocal scanning microscope (LSM 510 META; Zeiss, Germany).

### Western Blot

Frozen intestine samples were homogenized in an extraction buffer (1×4 w/v) containing a cocktail of protease inhibitors (Solarbio, Beijing, China), with the resulting lysates centrifuged at 10,000×g at 4°C for 10 min. The supernatants were collected, with protein concentrations determined by DC protein assay (Bio-Rad Laboratories, Hercules, CA, USA). Extract containing 50 µg protein was loaded on each lane in 5% SDS-PAGE gels. The polypeptides were separated by electrophoresis and further transferred into nitrocellulose membrane (Bio-Rad Laboratories). Nitrocellulose membranes were then incubated with the aforementioned antibodies to β-actin (1∶4000), PCNA (1∶200), Lgr5 (1∶200), Msi-1 (1∶250) and ephrin-B3 (1∶200). Bound proteins were visualized with HRP-conjugated goat anti-mouse or rabbit IgG (1∶20,000, Bio- Rad Laboratories), and the Western-Bright ECL (APG BIO-China, Shanghai, China). Immunoblot images were captured in an Omega-Lum G imaging system (APG BIO-China).

### Realtime fluorescence quantitative polymerase chain reaction (qPCR)

RNA extraction was carried out using the Trizol extraction kit according to manufacturer' s instruction (Life Technologies-China, Shanghai, China), with purified RNA samples stored at −70°C until use. cDNAs were produced by retrograde transcription using commercial kits including Accupower, RocketScript and RT PreMix (Bioneer-China, Shanghai, China). Specific DNA fragments were amplified using RealMasterMix (Probe) and miRcute miRNA with a Stratagene cycler (Tiangen Biomart, Beijing, China). The following forward (F) and reverse (R) primers were used for qPCR amplifications (synthesized by Bioneer-China). LGR5-F: 5′-TGCCATTATTCACCCCAAC-3′; LGR5-R: 5′-CACAGCACTGGTAAGCGTATG-3′; ephrin-B3-F: 5′-GCTGCTGTTAGGTTTTGCG-3′; ephrin-B3-R: 5′-CCCCGATCTGAGGGTAAAG-3′; Msi-1-F: 5′-AGGGGTTTCGGCTTCGT-3′; Msi-1-R: 5′-TGACCATCTTAGGCTGTGCTC-3′; PCNA-F:5′-GGTGAAGTTTTCTGCGAGTG-3′; PCNA-F: 5′-GGTGAAGTTTTCTGCGAGTG-3′; rat β-actin-F: 5′-GTCAGGTCATCACTATCGGCAAT-3′; and rat β-actin-R: 5′-AGAGGTCTTTACGGATGTCAACGT-3′.

### Imaging, data processing and statistic testing

H.E. stained sections were examined on an Olympus (BX60) fluorescent microscope equipped with a digital camera and image analysis system (CellSens, Olympus, Japan). Images (1600×1200 pixels) covering the entire intestine cross-section were taken using the 4× objective for morphometric analyses. The areas of the mucosal layer as well as the layer occupied by crypts were measured across the entire cross-sectional field of the intestine using the Image-J software, with the outside perimeter of the intestine also measured correspondingly. Fluorescent labeling was examined on a Leica DM500 Microscope using the 10× objective, with 10 non-overlapping microscopic fields taken randomly for each sample. Images were merged, and cells exhibiting PKH26 fluorescence (red) and those colocalizing PKH26 signal with immunoflourescence (green) were recorded for each merged image. These records were summed, and the mean rate of colocalization for a given maker was calculated for each case, and the each experimental animal group. Western blot images were analyzed by measuring optic densities of immunoblotted protein bands, which were normalized to the internal standard (β-actin) for statistical comparison. Similarly, qPCR data were normalized to internal reference (β-actin) before calculation of means. Statistical comparisons were carried out using one or two-way ANOVA with Bonferroni's multiple comparison (Prism GraphPad 4.1, San Diego, CA, USA). The minimal significance level was set at P<0.05. Figure panels were assembled using Photoshop 7.1.

## Results

### Evaluation of indomethacin-induced intestinal injury in rats

**Figure 1:**
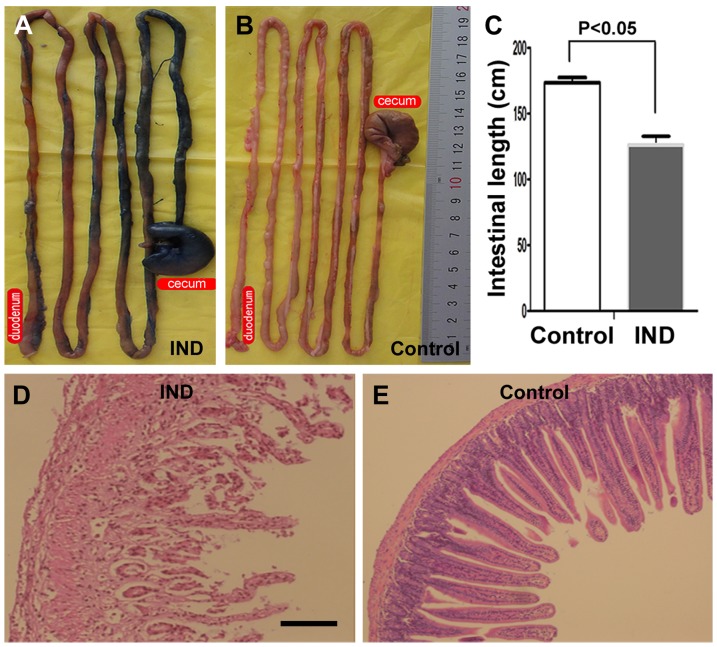
Evaluation of intestinal lesion after indomethacin administration. Panels (A) and (B) show that gross appearance of intestines from rats received indomethacin treatment (A) relative to control (B, saline-treated) surviving 4 days, as stained with Evans blue. The small intestines and appendix from the former appear in dark blue (A), whereas the intestine from healthy controls appears pink (B). The intestinal length is shortened in the indomethacin-treated rats relative to controls (C). In H. & E. preparation, the injured intestine show loss and disruption of the villi, the swelling of the intestinal wall, and the ulcerative lesion with fibrinoid plaques, edema, epithelial exfoliation, and granulocyte infiltration of leukocytes (D), relative to normal control (F). Scale bar  = 500 µm in (D) applying for (E).

We established a rat IBD model by indomethacin treatment in the present study [10]. To confirm the occurrence of GI injury, a group of animals (n = 4) were sacrificed 4 days after indomethacin administration (Fig. 1). By visual inspection, the small and large intestines of the indomethacin-treated rats appeared dark blue with 1% Evans blue stain (Fig. 1A), whereas the intestine of healthy control rats appeared pink (Fig. 1B). The total length of small intestine was shortened in the indomethacin-treated rats (127±9.5 cm) than normal controls (171.4±4.4 cm) (Student-t test, P<0.05). There was noticeable extension in the diameter of the small intestine in the indomethacin-treated group (Fig. 1A, B). Haematoxylin and eosin (H&E) stain demonstrated histological disruption, atrophy of mucosa, and thinning of villi, with ulcerative lesions penetrating the proper muscle layer. Edema, epithelial exfoliation and infiltration of leukocytes appeared to be also present in the indomethacin-treated intestine relative to control (Fig. 1D, E).

### Homing and differentiation of transplanted BMMSCs in injured rat intestine

Details of *in vitro* preparation, characterization and fluorescent labeling of BMMSCs were described in previous studies [11]. In the present study donor bone Fluorescence-activated cell sorting (FACS) analysis was used to confirm the expression of signature antigen markers of mesenchymal stem cells. Thus, 98.83% of the isolated cells expressed CD29; 98.22% expressed CD34, a myeloid progenitor cell antigen that is also present in endothelial cells and some fibroblasts; 34.88% expressed CD44, stem cell marker; and 85.30% expressed CD45, a hematopoietic and leukocyte marker [13]; 38.79% expressed CD90. Overall, the BMMSCs harvested from SD rat femur appeared to be largely bone marrow mesenchymal stem cells [14].

**Figure 2:**
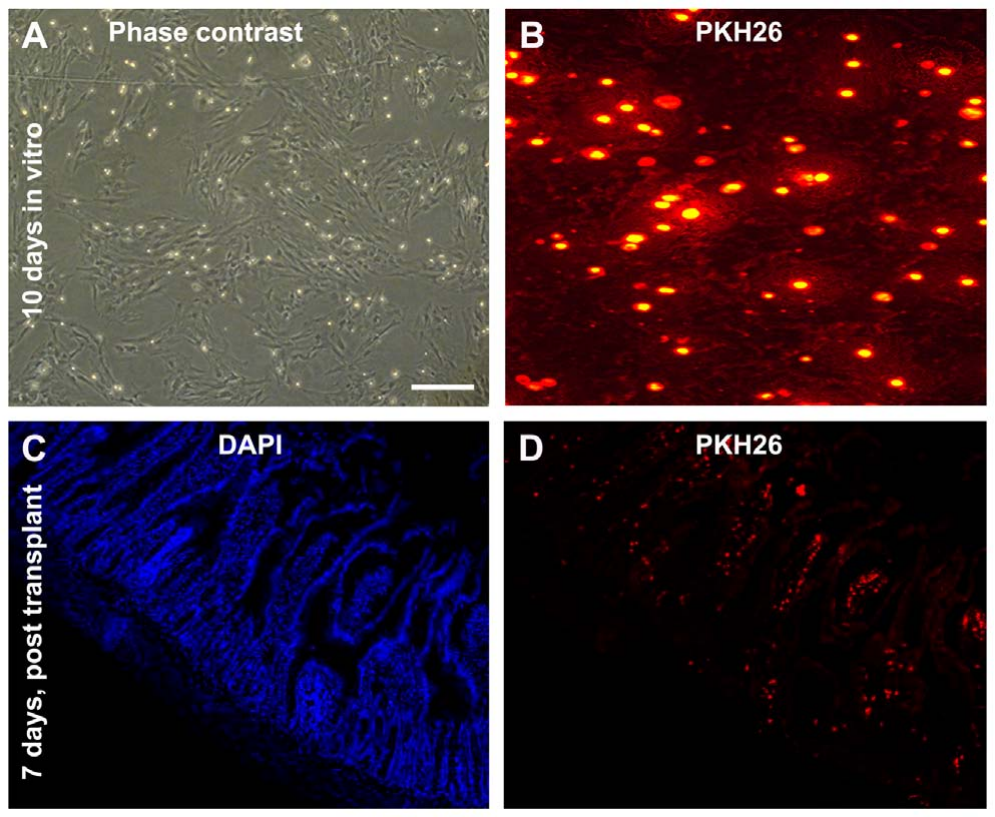
*In vitro* expansion and PKH26 labeling of bone marrow mesenchymal stem cells (BMMSCs) harvested from a donor rat, and homing of these labeled cells to the small intestine in recipient rat with indomethacin. (A) A large number of cells adhering to a culture dish with spindle-shape morphology at 10 days *in vitro*. (B) Culture cells pre-labeled by PKH26 show bright red fluorescence. Panels (C) and (D) show the distribution of PKH26 positive BMMSCs in the wall of the small intestine 7 days after infusion, with the cells mostly localized to the mucosal region. Scale bar  = 500 µm in (A) applying for (C, D), equivalent to 125 µm for (B).

PKH26 fluorescent labeling of BMMSCs was carried out following 10–15 days of *in vitro* expansion (Fig. 2A). The success of labeling for each batch of cells was confirmed under microscope (Fig. 2B) before *in vivo* application. Red fluorescent cells were could be detected *in situ* in the wall of small and large intestines for about 3 weeks post-transplantation. In normal intestinal tissue from naive control animals, only a few fluorescent cells were encountered (data not shown). In contrast, a considerable amount of red fluorescent cells was present in the injured intestines from indomethacin-treated rats, predominantly in the villi areas (Fig. 2C, D).

**Figure 3:**
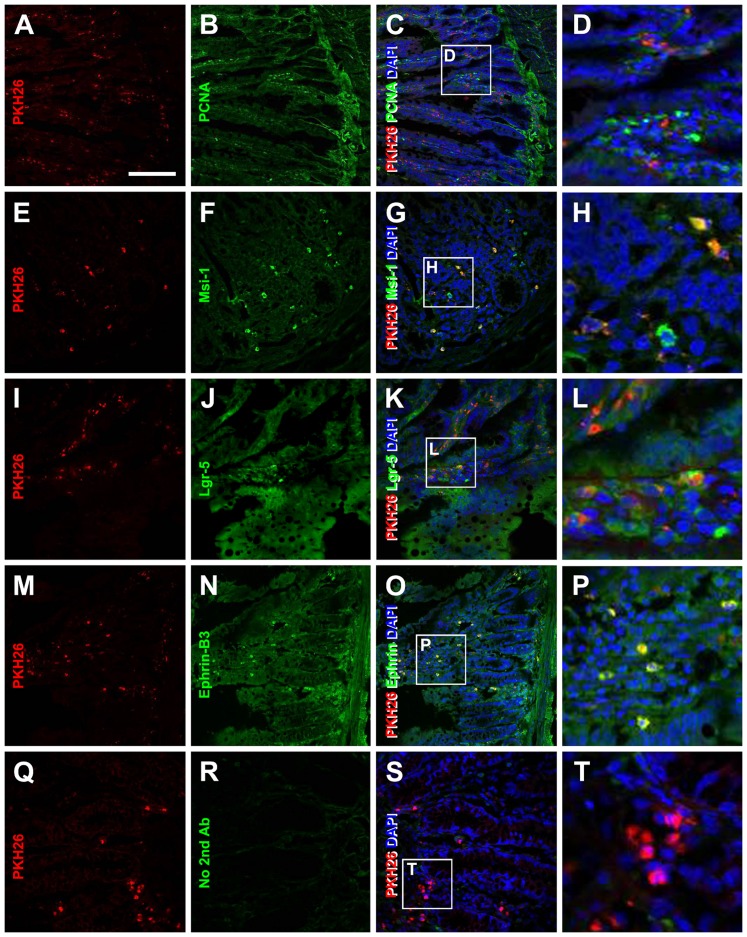
Fluorescent microscopic images showing potential *in situ* proliferation and transdifferentiation of transplanted BMMSCs in recipient rat small intestine with indomethacin injury. The left panels (A, E, I, M, Q) show PKH26 positive BMMSCs in the intestinal mucosa. These cells show partial colocalization with the endogenous proliferative cell nuclear antigen (PCNA) (B–D), and with molecular markers of mucosal stem cells or precursor including Msi-1 (E-H), Lgr5 (I–L) and Ephrin-3 (M–P). Panels (Q–T) show an example of negative assay control, indicating the lack of green fluorescence in the tissue by excluding the DyLight 488-conjugated secondary antibody in immunohistochemical preparation. Scale bar  = 250 µm in (A) applying to the 3 left panels, equivalent to 50 µm for the most right panels.

We evaluated a potential *in situ* proliferation of the transplanted BMMSCs in the GI tract at 2 and 3 weeks post cell transplantation by double labeling of PKH26-labeled cells with an endogenous proliferative marker, PCNA. PKH26-labeled cells were found to colocalize with PCNA at both surviving points (Fig. 3A–D). Cell counts were carried out in 4 animals surviving 2 weeks using 10 double-labelled small intestine sections per animal. PKH26-labeled cells were found to colocalize with PCNA at an estimated mean rate of 50.1±12.2% (137/235; 204/327; 131/354 and 114/267, respectively, for the 4 individual animals).

To explore the possibility of transdifferentiation of transplanted bone marrow cells into gut mucosal cells, we examined the colocalization of the PKH26-labeled cells with markers of immature crypt cells of the GI, including Msi-1, leucine-rich-repeat-containing-protein-coupled receptors (Lgr5) and ephring-B3 [15]–[17]. Subpopulations of PKH26-labeled cells were found to co-express Msi-1 (Fig. 3E–H), Lgr5 (Fig. 3I–L) and ephrin-B3 (Fig. 3M–P) in indomethacin-treated rats surviving 2 and 3 weeks after infusion of the PKH26-labeled bone marrow cells. Cell counts were performed in double-labelled small intestine sections from 4 indomethacin-treated animals surviving 3 weeks after cell transplant. PKH26-labeled cells were colocalized with Msi-1 at a measured mean rate of 53.3±14.5% (101/231; 119/304; 221/314 and 166/277, respectively, for the 4 individual animals, same format below); with Lgr5 at a measured mean rate of 45.2±7.5% (125/244; 102/253; 101/194 and 87/233); and with ephring-B3 with a mean rate of 58.3±9.2% (153/237; 112/217; 121/246 and 165/244).

### Effect of BMMSCs and SCF administration on intestinal mRNA expression

**Figure 4:**
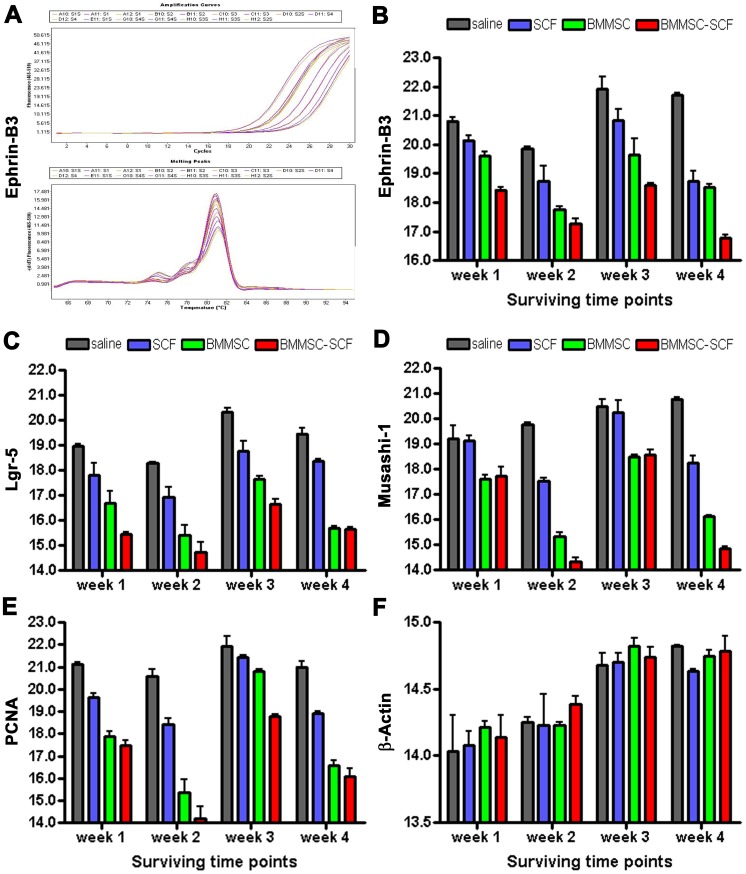
Effect of BMMSCs and stem cell factor (SCF) therapies on intestinal mRNA expression following indomethacin injury in rats. Panel (A) shows an example of the traces of real-time quantitative PCR (qPCR) for, with the top reflecting fluorescent signals vs amplification cycles and the ephrin-B3 bottom vs temperature changes. Panel (B) blots the means of fluorescent absorbent signals in the 4 animal groups at 4 surviving time points. Other panels (C–F) show data for the levels of mRNAs of additional markers as indicated. For details regarding statistical analyses, referring to the result section.

The mRNA levels of proliferative and mucosal molecular markers were determined by qPCR in intestinal tissues at 1, 2, 3 and 4 weeks after experimental treatments (Fig. 4). Levels of ephrin-B3 mRNA expression (expressed by fluorescent absorbent signal) were different among the groups by two-way ANOVA test (P = 0.002, F = 7.53, DF = 9, 3, 3, 32, same statistic test below), exhibiting treatment (P<0.0001, F = 63.4, DF = 3, 32) and time (P<0.0001, F = 22.3, DF = 3, 32) dependent overall differences. Post-hoc analysis indicated that there were differences between the saline group relative to each of the other groups at all time points except for the saline vs SCF groups at the 1 week point. The SCF vs BMMSCs groups showed statistically significant differences at the week 3 but other time point, whereas the SCF vs BMMSCs-SCF groups were different at all time points. The BMMSCs vs BMMSCs-SCF exhibited differences at the week 1, 3 and 4 time points (Fig. 4A, B). Levels of Lgr5 mRNA expression showed treatment (P<0.0001; F = 73.4; DF = 3, 32) and time (P<0.0001; F = 18.6; DF = 3, 32) dependent difference. Significant differences existed for the saline group relative to all other groups at all time points, for the SCF groups relative to the BMMSCs as well as the BMMSCs-SCF groups at all time points. For the BMMSCs vs BMMSCs-SCF groups, difference existed at the week 1 time point (Fig. 4C). Levels of Msi-1 mRNA expression showed an overall treatment (F = 56.8, P<0.0001) and time (F = 26.6, P<0.0001) dependent difference (P<0.0001, F = 13.6, DF = 9, 3, 3, 32). Post-hot tests indicated statistically significant differences between the saline vs BMMSCs groups, between the saline and BMMSCs-SCF groups, between the BMMSCs vs SCF groups, and between the SCF and BMMSCs-SCF groups, at all time points (P<0.0001 to P<0.001 for paired mean comparisons). For the BMMSCs in comparison with BMMSCs-SCF groups, significant differences existed at the week 2 and week 4 surviving points (Fig. 4D).

The levels of PCNA mRNA expression in small intestinal tissue lysate were also affected by BMMSCs and SCF treatments. There was an overall difference among the groups (P<0.0001, F = 7.27, DF = 9, 3, 3, 32), which related to significant treatment (P<0.0001; F = 57.0; DF = 3, 32) and time (P<0.0001; F = 32.5; DF = 3, 32) dependent variations. Post-hoc tests indicated significant differences between the saline group relative to each of other groups at all time points (P<0.05 to P<0.0001). The means between the SCF and BMMSCs groups were different at all (P<0.0001) but the week 3 time points. The means between the BMMSCs and BMMSCs-SCF groups were different at all time points (P<0.0001) (Fig. 4E). The mRNA levels of the β-actin housekeeping gene were assayed as an internal control for the qPCR quantifications of the above molecular markers. The means did not show an overall difference among the groups by two-way ANOVA (P = 0.95, F = 2.35, DF = 9, 3, 3, 32). There existed no treatment-dependent significant difference (P = 0.55, F = 1.59, DF = 3, 32), although there was a time-dependent difference (P<0.0001, F = 72.2, DF = 3, 32). However, Bonferroni post-tests indicated that there were no significant differences between each of the two treatment groups at any time point (Fig. 4F).

### Effect of BMMSCs and SCF administration on intestinal protein expression

**Figure 5:**
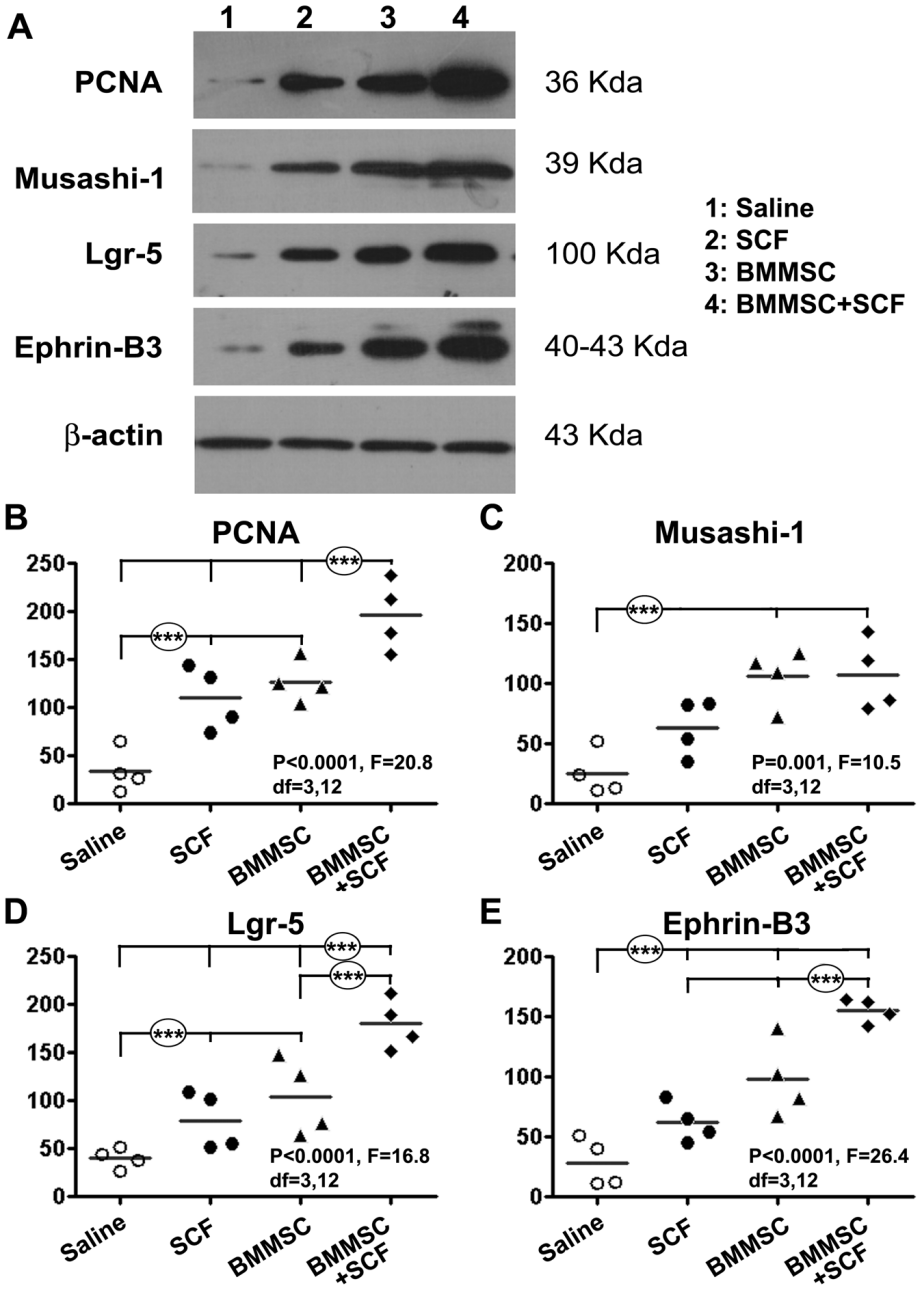
Effect of BMMSCs and stem cell factor (SCF) therapies on intestinal protein expression following indomethacin injury in rats surviving 4 weeks. Panel (A) shows western blot images of protein levels from one set of comparing groups of animals. Note the increased protein levels of PCNA, Msi-1, Lgr5 and ephrin-B3 relative to the internal control β-actin in the BMMSCs and SCF treated groups in comparison with saline group. The quantitative data and statistics for individual proteins are summarized as panels (B–E) as indicated. All data are expressed as the percent levels of β-actin in the same tissue lysate loads.

To further confirm an effect of the experimental treatments on the expression of proteins, we quantitatively assayed the PCNA and intestinal markers by western blot using tissues from 4 week surviving groups (Fig. 5A). Levels of PCNA protein were significantly different among the groups (P<0.001, F = 20.8, DF = 3, 12, one-way ANOVA), with Bonferroni's multiple comparison (post-hoc) indicating differences exciting between the saline group relative the SCF and the BMMSCs groups, and between the BMMSCs group relative to all other groups (Fig. 5B). Levels of Msi-1 protein were significantly different between the 4 groups (P = 0.001, F = 10.5, DF = 3, 12, one-way ANOVA). Post-hoc test indicated statistical difference between the saline group relative to the BMMSCs and BMMSCs-SCF groups (Fig. 5C). Levels of Lgr5 showed an overall difference among the groups (P = 0.0001, F = 16.8, DF = 3, 12, one-way ANOVA). Post-hoc test indicated statistically significant differences between the saline vs other groups, BMMSCs-SCF vs other groups, and the BMMSCs vs the BMMSCs-SCF groups (Fig. 5D). There were differences among the groups for the levels of ephrin-B3 protein (P<0.0001, F = 26.4, DF = 3, 12, one-way ANOVA). Specifically, statistically significant difference occurred between the saline relative to other groups, between the BMMSCs-SCF relative to the SCF and the BMMSCs groups (Fig. 5E).

### Improved histological recovery following BMMSCs transplant

**Figure 6:**
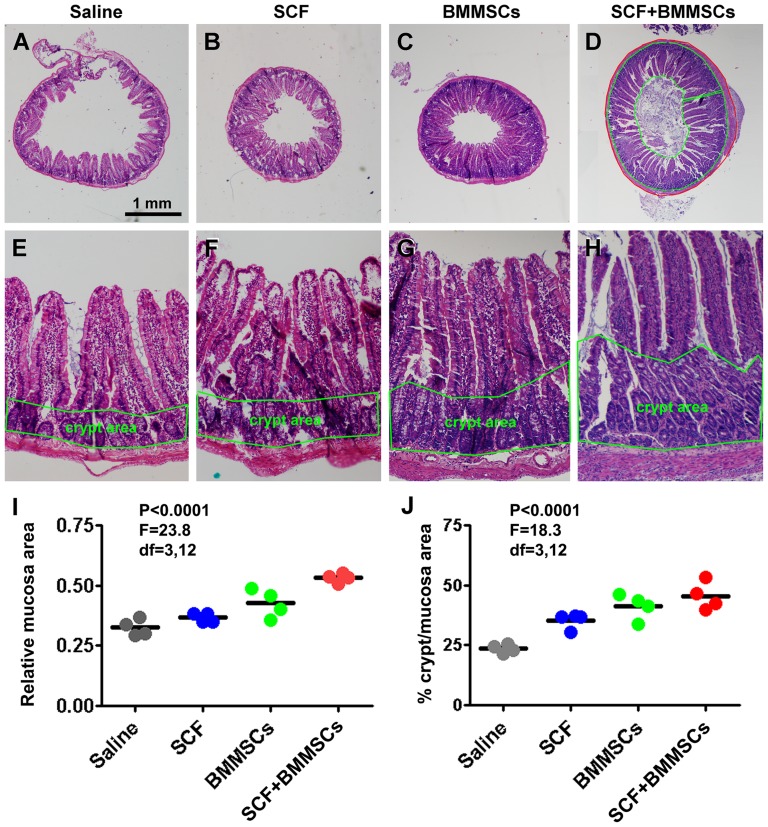
Effect of BMMSCs and stem cell factor (SCF) therapies on intestinal mucosal regeneration following indomethacin injury in rats examined at the 4 week surviving time point. Image panels (A–H) show representative low and high magnification H. & E. stain images of the small intestines from the 4 animal groups, sampled at the mid-point between beginning of the duodenum and appendix. The relative mucosal area is defined by the measured mucosal area divided by the outside perimeter of small intestine (as illustrated in panel D). There exists a trend of increase in the relative mucosal area in the BMMSCs and SCF treated groups, most evident in the BMMSCs and SCF co-treatment group (I). Relative area of the crypt layer to the total mucosal layer is defined by the ratio in the given intestinal cross-section (as illustrated in panel H). The means of the ration tend to increase in BMMSCs and SCF treated groups, especially in the BMMSCs and SCF co-treatment group (J). Scale bar  = 1 mm in (A) applying to (B–D), equivalent to 250 µm for (E–H).

Given that transplanted BMMSCs resided mostly in the mucosa (villi and crypts), we determined whether the therapies might improve the histological recovery of the injured intestine. This analysis was carried out in the 4 week surviving groups (Fig. 6). The cross-sectional mucosal areas and outside perimeters were obtained at 4 locations of the small intestine with equal distance (Fig. 6A–H). The ratio of mucosal area divided by perimeter was calculated for each sample, yielding an index of relative mucosa area (Fig. 6I). There was a significant difference in relative mucosal area among the groups (P<0.0001, F = 23.8, df = 3, 12, one-way ANOVA). Post-hoc test indicated a significant reduction of the relative mucosal area in the saline group relative to the BMMSCs and BMMSCs-SCF groups. The relative mucosal area was also significantly increased in the BMMSCs-SCF group relative to the SCF and the BMMSCs groups (Fig. 6I). Considering the crypts are the principal proliferative elements for mucosal regeneration, we further calculated the ratio of the area of occupied by the crypts relative to the total mucosal area in each sample, yielding means for individual animals and groups (Fig. 6E–H). The mean relative crypt to total mucosal areas tended to increase in the SCF and BMMSCs treatment groups relative to the saline group (Fig. 6J), showing significant difference by one-way ANOVA test (P<0.0001, F = 18.3, df = 3, 12). Specifically, Bonferroni's multiple comparison tests indicated statistical difference in the saline group relative to all other groups, and between the SCF and the BMMSCs-SCF groups (Fig. 6J).

## Discussion

Bone marrow stem cells may be used as a form of autograft, and are of potential therapeutic utility for tissue regeneration/organ repair. Evidence suggests that BMMSCs therapy facilitates tissue regeneration and improve functional recovery in a number of central and peripheral systems after acute or chronic injury [18], [19]. Bone marrow stem cell transplantation has been considered for the treatment of IBD and certain degenerative gastrointestinal disorders [20], [21]. For instances, previous studies have shown that transplanted BMMSCs labelled with BrdU, retrovirus or Y chromosome may home into the digestive tracts [22]. A few studies also suggest that transplanted bone marrow cells may colocalize with some gastrointestinal cell markers, such as Lgr5 and Ascl-2 [23].

In the present study, we observe that rats treated with indomethacin show gross anatomical appearance, histological changes and gastrointestinal symptoms of IBD. Thus, there is significant mucosal degeneration in the small and large intestines in the experimental rats as determined by histological evaluation. The height and area of the villi are dramatically reduced within a few days after indomethacin treatment. These histological and functional data are consistent with previous findings regarding the usefulness of indomethacin in inducing an experimental of model of IBD in rodents [10].

To explore the therapeutical potential of bone marrow stem cell transplant in IBD, BMMSCs are isolated from adult SD rats and expanded *in vitro* in the current study. The haemopoietic nature of the cultured cells is confirmed with a panel of specific membrane markers of bone marrow stem cells, including CD29, CD34, CD44, CD45 and CD90 [13]. The *in vitro* expanded BMMSCs are efficiently labelled with PKH26 in order to track these cells *in vivo* for a few weeks following transplantation.

In rats subjected to experimental IBD, transplanted PKH26-labeled cells are found to reside in the injured intestinal areas, mostly evidently in the crypts and villi. These cells show a considerable extent of colocalization with PCNA, implicating that they undergo *in situ* proliferation in the gut. Importantly, the transplanted bone barrow cells may co-express several molecular markers of precursors or immature intestine mucosal cells including Lgr5, Msi-1 and ephrin-B3. Lgr5 is expressed in precursors of intestinal secretory cells [24]. Msi-1 is an RNA-binding protein that has been shown to be a critical regulator of asymmetric division in stem cells. Msi-1 is expressed in both intestinal stem cells and their early progeny. Ephrin B3 is strongly expressed on cells at the bottom of the crypt, peaking at cell positions 4–6 (putative intestinal stem cells location). Thus, ephrin B3 is a useful cell surface marker to study intestinal stem cells [25]. Together, these histological and morphological data suggest that transplanted BMMSCs may home to in injured gut and transdifferentiate into stem-like intestinal stem cells in rats. The homing and early transdifferentiation of the transplanted BMMSCs in the injured gut appear to play a beneficial role in mucosal regeneration. Compared to untreated animals, BMMSCs transplantation leads to increases in the overall thickness of the mucosa as well as the relative areas of the crypts in the intestine. The crypts are considered the proliferative niche for mucosal renewal. Therefore, our findings support the notion that bone marrow stem cell therapy may be useful in the treatment of inflammatory bowl diseases [26], [27].

Certain soluble growth factors may play a critical role for mobilization, survival and successful integration of circulating multipotent stem cells in the target organs/tissue, which may be also of value in stem cell therapy for degenerative diseases [28], [29]. Adjunctive use of stem cell trophic factors has been shown to improve the outcome of BMMSCs therapy in a number of disease conditions, therefore are recommended in clinical trials of stem cell therapy. In the present study we demonstrate that the stem cell factor alone can improve the intestinal mucosal regeneration following indomethacin-induced injury for a certain degree. Co-administration of this factor appears to exhibit a synergistic beneficial effect on BMMSCs transplant to intestinal recovery. This effect includes a stimulation of the expression of cell proliferative antigen (PCNA) as well as particular molecules of mucosal stem cells or precursors including Msi-1, Lgr5 and ephrin-B3. Furthermore, mucosal and crypt areas are increased to a greatest extent in the BMMSCs-SCF treatment group after intestinal injury.

## Conclusions

The present study shows *in vitro* expanded BMMSCs relocate to but exhibit local proliferation in the injured intestinal tissues in a rat model of IBD. They also appear to transdifferentiate into intestinal mucosal cells during the first few weeks post-transplantation. The BMMSCs therapy is associated with improved mucosal regeneration in indomethacin injured rat intestines. Importantly, adjunct administration of stem cell factor facilitates the beneficial role of bone marrow stem cell therapy in intestinal mucosal regeneration in this model of IBD.
